# Atypical Presenting Symptoms of Acute Onset Systemic Lupus Erythematosus with Enteritis and Cystitis

**DOI:** 10.1155/2016/8579812

**Published:** 2016-03-15

**Authors:** Mayu Yagita, Kohei Tsujimoto, Masato Yagita, Masaaki Fujita

**Affiliations:** Department of Clinical Immunology and Rheumatology, The Tazuke-Kofukai Medical Research Institute, Kitano Hospital, Osaka 530-8480, Japan

## Abstract

Lupus enteritis and lupus cystitis are relatively rare manifestations of systemic lupus erythematosus. Some patients develop severe complications such as bowel perforation, infarction, obstruction, or irreversible bladder dysfunction. Early diagnosis is critical for management of lupus enteritis and cystitis. We report a 48-year-old Japanese man who presented with initial manifestations of abdominal pain, severe diarrhea, and bloody feces. The diagnosis was delayed due to atypical initial symptoms, resulting in clinical worsening. Physicians should be aware of typical computed tomography findings of lupus enteritis and lupus cystitis.

## 1. Introduction

Systemic lupus erythematosus (SLE) is a systemic chronic inflammatory disease. While fever, joint pain, and rash are the most common symptoms, some patients predominately show manifestations in the hematologic, renal, or central nervous system. This lack of typical presentation often causes delay in diagnosis of SLE. Although lupus enteritis is relatively uncommon, it may lead to life-threatening complications such as bowel perforation [1, 2]. Early diagnosis is of great importance in management of lupus enteritis. Here, we describe a patient who presented with lupus enteritis and lupus cystitis as initial manifestations of SLE, for whom the diagnosis was delayed due to the atypical presentation.

## 2. Case Report

**Figure 1:**
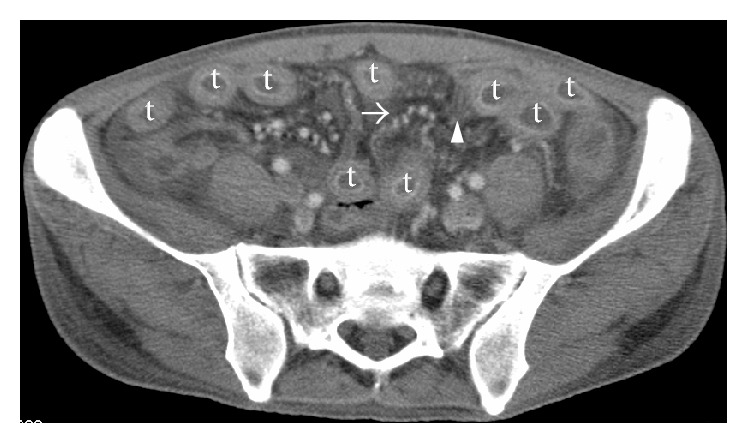
CT scan reveals bowel wall thickening and enhancement and engorgement of mesenteric vessels. Target sign (t), comb sign (arrow head), and nodular sign (arrow).

**Figure 2:**
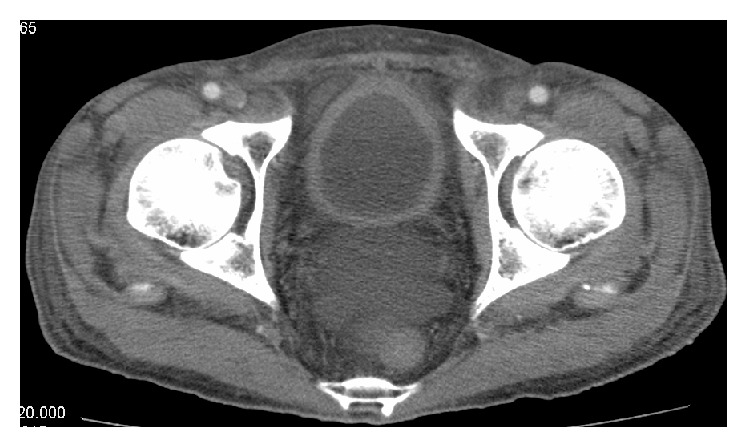
CT scan reveals bladder wall thickening.

On July 14, 2015, a 48-year-old Japanese man was admitted to an outside hospital with fever, vomiting, intermittent abdominal pain, severe diarrhea, and bloody feces. Laboratory test results were as follows: white blood cell count, 2200/*μ*L; hemoglobin, 14.9 g/dL; platelets, 14.9 × 10^4^/*μ*L; C-reactive protein (CRP), 21.2 mg/dL; amylase, 529 U/L (normal < 120 U/L); albumin, 3.1 g/dL (normal > 3.8 g/dL); urea nitrogen, 48 mg/dL; creatinine, 1.33 mg/dL; and urine protein, 1+. Abdominal computed tomography (CT) showed bowel wall thickening and enhancement. A colonoscopy identified edematous changes from the ileum to the rectum. Colon biopsy showed lymphocyte infiltrations in the mucous membrane covering the epithelial cells. These findings led to a diagnosis of severe infectious enteritis. With antibiotics and bowel rest, diarrhea and abdominal pain partially improved. On August 5, oral intake was resumed. However, the gastrointestinal symptoms recurred, and abdominal CT still showed edematous bowel wall. It was thought unlikely that the symptoms were caused by infectious enteritis alone. On August 20, he was referred to us in the Department of Clinical Immunology at Kitano Hospital, and his symptoms were reevaluated. On admission, no alopecia, rash, oral ulcers, arthritis, neurological disorder, or bladder irritation was noted. Physical examination revealed tenderness in the epigastrium and diminished bowel sounds. Laboratory test results were as follows: anti-nuclear antibody (indirect immunofluorescence test) 1 : 160 (normal < 1 : 40); anti-dsDNA antibody 55.8 IU/mL (normal < 12 IU/mL); CH50 24.2 U/mL (normal: 30–50 U/mL); C3 88 mg/dL (normal: 65–135 mg/dL); C4 32 mg/dL (normal: 13–35 mg/dL); and urine protein 0.39 g/gCr. Chest CT showed bilateral pleural effusions. The patient fulfilled both the Systemic Lupus International Collaborating Clinics (SLICC) classification criteria and the American College of Rheumatology revised criteria (serositis, lymphopenia, positive anti-ANA antibody, and positive anti-dsDNA antibody) [1, 2]. Based on these findings, the patient was diagnosed with SLE developed with lupus enteritis. Intravenous administration of methylprednisolone (100 mg/day) was started immediately. However, 2 weeks later abdominal CT still showed bowel wall thickening and enhancement (Figure 1: target sign), engorgement of mesenteric vessels (Figure 1: nodular sign and comb sign), bladder wall thickening (Figure 2), ascites, and bilateral pleural effusions. He was treated with intravenous methylprednisolone (1000 mg) for 3 days, followed by intravenous methylprednisolone (100 mg/day). Bowel wall thickening rapidly improved, and the ascites and bilateral pleural effusions disappeared. The steroid was gradually tapered and changed to oral administration. However, his gastrointestinal symptoms recurred when the oral prednisolone dose was 40 mg/day (0.8 mg/kg/day). Intravenous cyclophosphamide at 1100 mg (750 mg/mm^2^ BSA) was administered. He was discharged on oral prednisolone (35 mg/day) followed by monthly intravenous cyclophosphamide without recurrence.

## 3. Discussion

Lupus enteritis is typically steroid-responsive with prompt treatment [3, 4]. However, some patients develop severe complications such as bowel perforation, infarction, or obstruction. Early diagnosis is critical for management of lupus enteritis. However, clinical symptoms of lupus enteritis are often nonspecific with abdominal pain, diarrhea, and bloody feces. Unless a patient presents with typical symptoms of SLE, such as fever, rash, joint pain, and/or hematologic, renal, or central nervous system manifestations, diagnosis can often be delayed. The most useful tool for the diagnosis of lupus enteritis is abdominal CT [4, 5]. Typical CT findings of lupus enteritis include bowel wall thickening with enhancement (target sign), engorgement of mesenteric vessels (nodular sign and comb sign), and increased attenuation of mesenteric fat [4, 6, 7]. Also, lupus enteritis and lupus cystitis often occur concurrently; the common mechanism is thought to be smooth muscle dyskinesia due to immune complex-mediated damage [8, 9]. Therefore, it is necessary to evaluate involvement of the urinary tract if lupus enteritis is suspected. In our patient, bowel wall thickening with enhancement (target sign), engorgement of mesenteric vessels (nodular sign and comb sign), and bladder wall thickening were also identified by retrospective analysis of the first abdominal CT. Physicians should be aware of the typical CT findings of lupus enteritis (target sign, nodular sign, and comb sign) and lupus cystitis (bladder wall thickening), although these findings are not specific to lupus enteritis as the above described abnormalities can also be seen in patients with inflammatory bowel disease or peritonitis [4, 5].

Several studies evaluated predictive factors for the recurrence of lupus enteritis. Koo et al. [10] have suggested that involvement of the colon and urinary tract may be associated with the recurrence of lupus enteritis. Kim et al. [11] and Yuan et al. demonstrated that bowel wall thickening (>8 or 9 mm) was a risk factor for recurrence. In addition, Yuan et al. showed that lymphopenia, hypoalbuminemia, and elevated serum amylase were associated with severe adverse events. Consistent with these findings, our case had involvement of the colon and urinary tract, bowel wall thickening (10 mm), lymphopenia, hypoalbuminemia, and elevated serum amylase, suggesting the patient was at high risk for recurrence and severe adverse events. As intravenous cyclophosphamide is recommended for patients with severe or steroid-resistant lupus enteritis [4, 5, 8, 9], our case was successfully treated with intravenous cyclophosphamide. However, corticosteroids might have been adequate for the management of lupus enteritis if the case had been promptly diagnosed. Early diagnosis is of great importance for the optimal management of lupus enteritis.
